# Next Generation Sequencing Based Transcriptome Analysis of Septic-Injury Responsive Genes in the Beetle *Tribolium castaneum*

**DOI:** 10.1371/journal.pone.0052004

**Published:** 2013-01-09

**Authors:** Boran Altincicek, Abdelnaser Elashry, Nurper Guz, Florian M. W. Grundler, Andreas Vilcinskas, Heinz-Wilhelm Dehne

**Affiliations:** 1 Rheinische Friedrich-Wilhelms-University of Bonn, INRES-Phytomedicine, Nussallee 9, Bonn, Germany; 2 Department of Plant Protection, Faculty of Agriculture, Ankara University, Ankara, Turkey; 3 Justus-Liebig-University of Giessen, Institute of Phytopathology and Applied Zoology, Giessen, Germany; National Institutes of Health, United States of America

## Abstract

Beetles (Coleoptera) are the most diverse animal group on earth and interact with numerous symbiotic or pathogenic microbes in their environments. The red flour beetle *Tribolium castaneum* is a genetically tractable model beetle species and its whole genome sequence has recently been determined. To advance our understanding of the molecular basis of beetle immunity here we analyzed the whole transcriptome of *T. castaneum* by high-throughput next generation sequencing technology. Here, we demonstrate that the Illumina/Solexa sequencing approach of cDNA samples from *T. castaneum* including over 9.7 million reads with 72 base pairs (bp) length (approximately 700 million bp sequence information with about 30× transcriptome coverage) confirms the expression of most predicted genes and enabled subsequent qualitative and quantitative transcriptome analysis. This approach recapitulates our recent quantitative real-time PCR studies of immune-challenged and naïve *T. castaneum* beetles, validating our approach. Furthermore, this sequencing analysis resulted in the identification of 73 differentially expressed genes upon immune-challenge with statistical significance by comparing expression data to calculated values derived by fitting to generalized linear models. We identified up regulation of diverse immune-related genes (e.g. Toll receptor, serine proteinases, DOPA decarboxylase and thaumatin) and of numerous genes encoding proteins with yet unknown functions. Of note, septic-injury resulted also in the elevated expression of genes encoding heat-shock proteins or cytochrome P450s supporting the view that there is crosstalk between immune and stress responses in *T. castaneum*. The present study provides a first comprehensive overview of septic-injury responsive genes in *T. castaneum* beetles. Identified genes advance our understanding of *T. castaneum* specific gene expression alteration upon immune-challenge in particular and may help to understand beetle immunity in general.

## Introduction

Parasites reduce the fitness of their hosts and therefore numerous host mechanisms have evolved to limit infectious diseases. In animals, the risk of an infection is reduced by physical and chemical barriers, by behavioral defense reactions such as avoidance or hygiene [1], and by the complex and highly evolved immune defense system. In vertebrates, the immune system is composed of the adaptive immunity including specific T-cell receptors and B-cell-derived antibodies and the evolutionarily more ancient innate immunity [2], [3]. Of note, vertebrate innate immunity shows many parallels to the invertebrate immunity. Insects, e.g. *Drosophila melanogaster*, have widely been used to elucidate invertebrate immune reactions. These reactions include entrapment of invading pathogens in clots, phagocytosis by immune-competent cells (hemocytes), and the induced production of antimicrobial peptides as well as reactive oxygen species, both underlying the induced expression of a wide array of immune-related genes [4]–[9].

The recent determination of the *Tribolium castaneum* genome sequence [10] enabled the identification of numerous immune-related genes by both homology-based [11] and experimental approaches [12]. These studies provided first important insights into the *T. castaneum* immunity; however, our understanding of *Tribolium* immune responses is still fragmentary. The expression levels of only a limited number of *Tribolium* genes have been determined upon immune-challenge [11], [12]. To gain deeper insights into *Tribolium* immune responses, here, we investigated the whole transcriptome of naïve and immune-challenged beetles by Illumina/Solexa next generation sequencing. To induce strong immune responses in *T. castaneum* we used a commercially available crude lipopolysaccharide (LPS) preparation derived from *Escherichia coli*, which has widely been used as an elicitor of immune responses in numerous vertebrates and invertebrate species [12]–[16].

The present sequencing approach resulted in the identification of the transcriptome of *T. castaneum* and the identification of 70 genes with significantly elevated and 3 genes with reduced mRNA levels upon septic injury as determined by fitting the expression data with generalized linear models.

## Materials and Methods

### Biological samples for transcriptional analysis

The *Tribolium* stock that we used in this study was the *T. castaneum* wild-type strain San Bernardino. In contrast to the genome-sequenced GA-2 *T. castaenum* strain, the strain San Bernardino is “wild-type” since no consecutive generations of virgin single-pair, full-sib inbreeding were performed for 20 generations to obtain near-homozygous inbred condition needed for proper genome-sequencing [10]. Beetles were maintained on whole-grain flour with 5% yeast powder at 31°C in darkness. For the experimental treatments, we have first randomly selected 40 young adult beetles (1–2 weeks after final ecdysis), which were subsequently divided by chance into two groups. LPS-challenge of 20 beetles was performed by ventrolaterally pricking of the imagoes abdomen using a dissecting needle dipped in an aqueous solution of 10 mg/ml lipopolysaccharide (LPS, purified *Escherichia coli* endotoxin 0111:B4, Cat. No.: L2630, Sigma, Taufkirchen, Germany), as described [12]. At eight hours post LPS-challenge treated beetles and a biologically independent sample of 20 unstabbed, but similar handled beetles (control) were frozen in liquid nitrogen. We extracted total RNA from frozen beetles using the TriReagent isolation reagent (Molecular Research Centre, Cincinnati, OH, USA) according to the instructions of the manufacturer and synthesized cDNA samples using oligio-d(T) primers with the SMART PCR cDNA Synthesis Kit (Clontech, Mountain View, CA, USA) as previously described [12]. Sequencing was done by the GATC GmbH (Konstanz, Germany) sequencing company on an Illumina GA2 sequencer.

### Data analysis and bioinformatics

We have deposited the short read sequencing data with the following SRA accession numbers at NCBI sequence database: SRX022010 (immune-challenged beetles) and SRX021963 (naïve beetles). Sequencing reads were mapped by the sequencing company with ELAND Illumina software using the first 32 bp with highest sequencing quality and score values over 30 indicating 99.9% accuracy [17] and allowing one mismatch to the reference sequence of the *Tribolium* genome sequencing [18]. To calculate statistical differences of the expression levels of genes between treatment and control and thereby to identify immune-responsive genes we utilized DESeq package [19] within Bioconductor [20] and R [21]. DESeq was used to normalize the count data, calculate mean values, fold changes, size factors, variance and P values (raw and adjusted) of a test for differential gene expression based on generalized linear models using negative binomial distribution errors.

### Identification of Single Nucleotide Polymorphisms (SNPs) and Deletion Insertion Polymorphisms (DIPs) and *de novo* assembly

Single Nucleotide Polymorphisms (SNPs) and Deletion Insertion Polymorphisms (DIPs) detection tools within the CLC genomic workbench (version 4.9) were used to determine sequence variants. First, all Illumina reads were prepared by trimming of ambiguous nucleotides (>2 N) and low quality bases (<0.05). First we mapped all reads against the Glean assembly transcripts. Then, the level of SNPs and DIPs quality and significance was assessed by adjusting the quality filter to select only SNPs and DIPs that exists in a window of at least 11 bases and does not score more than 2 gaps or mismatches. The quality of the central base of each window was set to be at least 20 and the surrounding bases at least 15. The significance filter was adjusted to ignore SNPs and DIPs that have a coverage less than 4 and variant level less than 35% of corresponding reads. *De novo* assembly has been performed with the CLC genomics workbench (version 4.9) with the *de novo* assembly algorithm for Illumina reads with default parameters settings (Min. similarity allowed = 0.8 at length fraction = 0.5, deletion and insertion cost = 3, and mismatch cost = 2).

### Sequence annotation

Sequence homology searches of predicted reference gene sequences (gleans) and subsequent functional annotation by gene ontology terms (GO), InterPro terms (InterProScan, EBI), enzyme classification codes (EC), and metabolic pathways (KEGG, Kyoto Encyclopedia of Genes and Genomes) were determined using the BLAST2GO software suite v2.3.1 [22]. Homology searches were performed remotely on the NCBI server through QBLAST: sequences were compared with the NCBI non-redundant (nr) protein database and matches with an E-value cut-off of 10^−3^, with predicted polypeptides of a minimum length of 15 amino acids, were scored. Subsequently, GO classification, including enzyme classification codes and KEGG metabolic pathway annotations, were generated. For final annotation, InterPro searches on the InterProEBI web server were performed remotely by utilizing BLAST2GO.

## Results and Discussion

### Mapping Illumina sequencing reads to predicted gene models of *T. castaneum*

To gain insights into *Tribolium* immune responses, we investigated the whole transcriptome of naïve and immune-challenged beetles by Illumina/Solexa next generation sequencing. This sequencing approach resulted in over 9.7 million cDNA reads with over 700 million bp sequence information and estimated 30× transcriptome coverage. About 3.8 and 4.0 million reads of Illumina sequencing of control and LPS-challenged animals, respectively, were mapped to predicted gene models of *T. castaneum*, which were built on the 3.0 genome assembly [10] (Table 1). We found that 11,679 predicted genes were expressed in both naïve and LPS-challenged adult *Tribolium* beetles. Additional sequences corresponding to the expression of further 642 and 739 predicted genes in naïve and LPS-challenged beetles, respectively, were also observed. In total, this approach resulted in the expression validation of 13,060 genes, representing almost 80% of the in total 16,422 predicted genes.

**Table 1:** Summary statistics for *Tribolium castaneum* transcriptome sequencing analysis.

	Illumina sequencing of control animals	Illumina sequencing of LPS-challenged animals
Total number of reads	4,626,793	5,120,575
Read length (bases)	72	72
Reads mapped to predicted gene models	3,829,729	4,004,894
Reads not mapped to predicted gene models but to ESTs or genome sequences	551,696	814,883
Reads not mapped	245,368	300,798

### Evidence for the need of gene model curation and identification of single-nucleotide polymorphisms (SNPs) and DIPs (short deletion and insertion polymorphisms)

About 14% of all sequencing reads could be assigned to published *T. castaneum* EST sequences or the genome sequence but not to predicted gene models indicating that several exons or genes might be miss-predicted in the current genome annotation. Therefore, we shared the present sequencing data with the beetleBase [23] and the iBeetle consortium [24], which are currently working on a next, more precise genome annotation. In addition, we identified over 155,000 positions of high quality single-nucleotide polymorphisms (SNPs) and 895 DIPs (short deletion and insertion polymorphisms) within the coding gene sequences between the *T. castaneum* strain San Bernardino used in the present analysis and the genome-sequenced strain Georgia GA-2 [10] (Table S1). This information might be helpful in future comparative studies investigating the potential impact of SNPs and DIPs on varying ecological traits of diverse *T. castaneum* strains. Furthermore, we performed a *de novo* assembly (Data S1), which might be helpful for future studies investigating e.g. alternative splicing events.

Interestingly, about 5% of all sequencing reads did not map to *T. castaneum* sequences but to sequences from other organisms such as the bacteria *Escherichia coli*, *Bacillus subtilis*, or *Azotobacter vinelandi*. These bacterial species may represent part of the beetle flora.

### Validation of present Illumina sequencing approach by comparing estimated fold change expression values with recently reported values determined by qRT-PCR analysis

**Figure 1:**
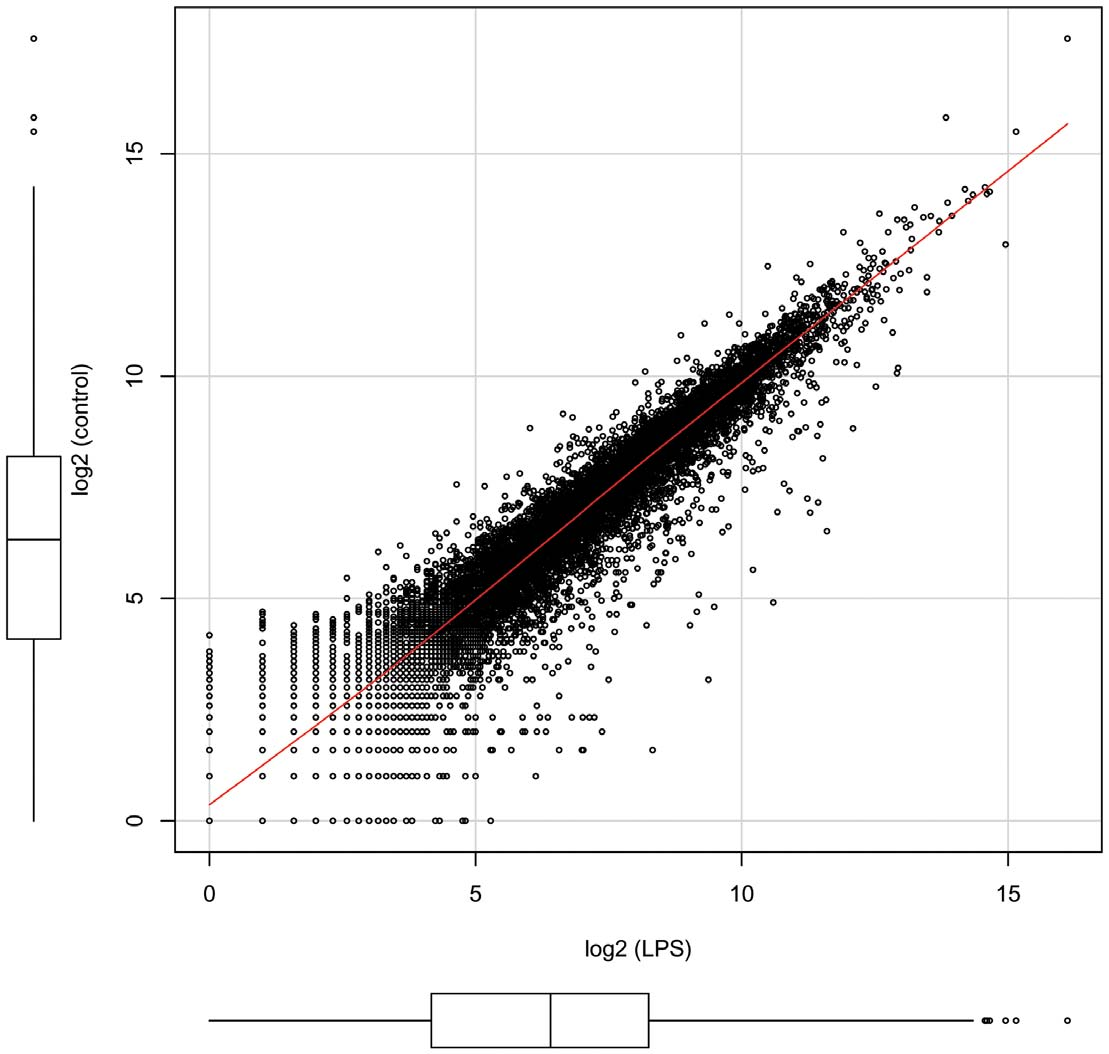
Gene expression in naive and immune-challenged beetles. All reads were aligned to predicted genes and are shown as log2 values derived from cDNA of naïve and LPS-challenged animals, respectively. The linear correlation is indicated by a red line (F-test, P, <2.2×10^−16^).

**Figure 2:**
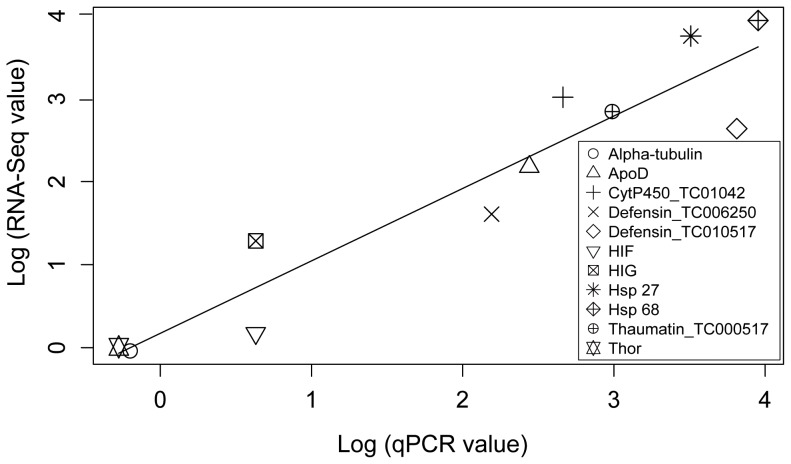
Correlation of gene expression levels of selected genes by both our recent qRT-PCR [**12**] and present RNASeq approach. The determined values of the expression levels of selected genes are shown as logN values. The values of both experiments were comparable and correlated with statistical significance (Pearson correlation, P, 0).

To determine differentially expressed genes between naive and LPS-challenged beetles we first checked whether sequencing samples were comparable. We counted the amount of reads aligned to predicted genes using only the first 32 bp of reads with highest sequencing quality and score values over 30 indicating 99.9% sequence accuracy [17] (Figure S1). In both treatments, we found that almost all genes were expressed at identical levels resulting in a significant linear correlation of the logarithmically transformed expression values (Figure 1). The regression analysis resulted in an adjusted R-squared value of 0.9073 (F, 1.143×10^5^; d.f., 11,677, P, <2.2×10^−16^). However, as expected, several potentially immune-responsive genes showed variance in their expression levels and we compared their expression rates with recently investigated immune-responsive genes [12]. Validating our present approach, the expression values determined by our recent qRT-PCR analysis of the house-keeping gene α-tubulin as control and several antimicrobial peptides such as defensins and thaumatin as well as stress-responsive genes such as heat shock factors [12] were found to be comparable to the values determined by the present RNA-Seq approach (Table 2). We found that the values of both experiments were highly similar and correlated with statistical significance (Pearson correlation factor of 0.95 of logarithmically transformed values with a Holm's method adjusted P values = 0) (Figure 2).

**Table 2:** Comparison of RNA level estimation by our recent qRT-PCR analysis [12] and present transcriptome sequencing approach.

Gene name	GLEAN_ID	Locus tag number_ID	Mean fold change value by qRT-PCR	Mean fold change value by RNA-Seq
Alpha-tubulin	GLEAN_04873	TcasGA2_TC004873	0.82	0.96
Defensin_TC010517	GLEAN_10517	TcasGA2_TC010517	45.26	14.00
Defensin_TC006250	GLEAN_06250	TcasGA2_TC006250	8.95	5.00
Thaumatin_TC000517	GLEAN_00517	TcasGA2_TC000517	19.88	17.18
ApoD	GLEAN_15563	TcasGA2_TC015563	11.48	8.84
Hsp 68	GLEAN_10172	TcasGA2_TC010172	52.12	51.50
Hsp 27	GLEAN_05338	TcasGA2_TC005338	33.42	42.67
CytP450_TC01042	GLEAN_10423	TcasGA2_TC010423	14.33	20.43
Thor	GLEAN_06808	TcasGA2_TC006808	0.76	1.01
HIG	GLEAN_03997	LOC661203	1.88	3.62
HIF	GLEAN_13241	LOC655772	1.88	1.19

### Identification of significantly induced or repressed genes upon LPS-challenge in *T. castaneum*

**Figure 3:**
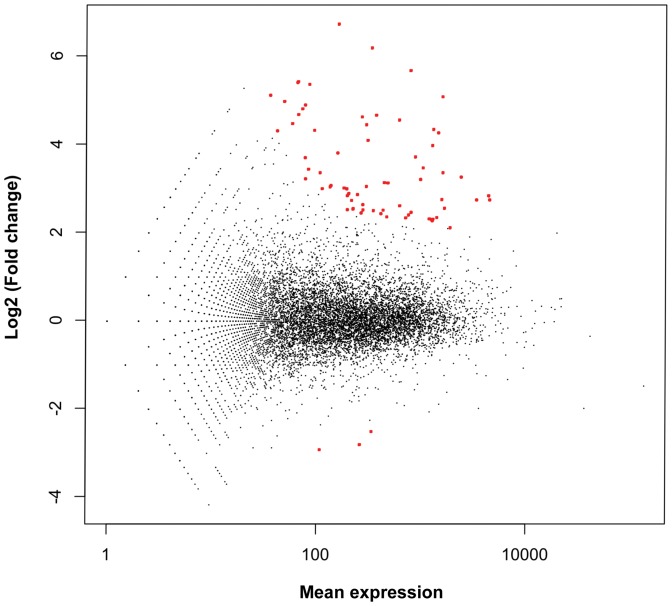
Significance plot. The log2 fold change value of each gene is shown against its base mean value. Differentially expressed genes with statistically significant difference at 5% FDR are indicated by red coloring.

To identify novel immune-responsive genes we calculated statistical differences of the expression levels between treatments utilizing DESeq package within Bioconductor and R. This powerful tool estimated the variance in our data and tested for differential gene expression [19]. Since the two biological independent samples from control and treated beetles resulted in comparable expression values (F, 1.143×10^5^; d.f., 11,677, P, <2.2×10^−16^), we took the variance estimated from comparing their count rates across conditions as described in the DESeq manual [25]. This analysis to identify differentially expressed genes is appropriate and will only cause the variance estimate to be too high, so that the test will err to the side of being too conservative [25]. We further used pools of 20 individuals per sample to average across biological replicates of individuals. In sum, normalized count data were fitted with a generalized linear model (GLM) estimating a negative binomial distribution to the calculated mean values of the two biologically independent samples with each containing pooled cDNAs of 20 individual beetles. Then the P values were adjusted for multiple testing with the Benjamini-Hochberg procedure, which controls false discovery rate (FDR) (Table S2). Finally, we obtained the statistically significant up-regulation of 70 genes and down-regulation of 3 genes with a 5% FDR (Figure 3).

To assign the potential functions of identified genes we performed an annotation step with blast2go (Table S3) and summarized differentially expressed genes (Table 3). We observed the strongly induced expression of numerous genes including specific serine proteases, Toll receptor, or cathepsin L that are reportedly immune-responsive also in *Drosophila* flies [6], [26]. Moreover, we found several genes encoding proteins with leucine-rich-repeat domains potentially involved in immune signaling reactions in *Tribolium*, which have not been investigated yet. The leucine-rich repeat domain is a common structural motif for the molecular recognition of microbes, which is also present in the prominent Toll-like receptors, evolutionarily conserved receptors initiating signaling reactions in animal immunity [2].

**Table 3:** Transcripts with significant differential expression upon LPS-challenge in adult beetles.

GLEAN_ID	Description	Fold change of expression	P value	FDR-adjusted P value
GLEAN_02785	serine protease P40	105.45	0.0000	0.0000
GLEAN_09706	heat shock protein 68a	72.49	0.0000	0.0000
GLEAN_10172	similar to heat shock protein 70	50.75	0.0000	0.0000
GLEAN_04540	hypothetical protein TcasGA2_TC004540	42.71	0.0000	0.0006
GLEAN_05338	similar to small heat shock protein 21	42.05	0.0000	0.0006
GLEAN_09776	similar to juvenile hormone-inducible protein with protein kinase domain	40.90	0.0000	0.0001
GLEAN_03541	similar to lethal(2)essential for life protein, l2efl	34.49	0.0001	0.0280
GLEAN_05951	hypothetical protein TcasGA2_TC005951	33.64	0.0000	0.0000
GLEAN_00067	similar to putative glutathione s-transferase	31.21	0.0000	0.0084
GLEAN_16345	hypothetical protein TcasGA2_TC016345 with chitin binding domain	29.57	0.0000	0.0006
GLEAN_09362	cathepsin L precursor	27.79	0.0000	0.0010
GLEAN_11793	similar to annexin IX CG5730-PC	25.43	0.0000	0.0022
GLEAN_13480	similar to dopa decarboxylase	25.17	0.0000	0.0000
GLEAN_13679	cytochrome P450-like protein	24.50	0.0000	0.0000
GLEAN_15598	Major Facilitator Superfamily transport protein	23.30	0.0000	0.0000
GLEAN_07154	Ets-domain transcription factor	22.08	0.0000	0.0083
GLEAN_11632	similar to inner membrane proteins	21.64	0.0000	0.0000
GLEAN_10423	cytochrome P450-like protein	20.13	0.0000	0.0000
GLEAN_09775	similar to Juvenile hormone-inducible protein with protein kinase domain	19.82	0.0000	0.0008
GLEAN_11074	short peptide	19.71	0.0002	0.0411
GLEAN_04539	leucine-rich repeat receptor-like protein kinase	19.06	0.0000	0.0000
GLEAN_00517	similar to antifungal thaumatin-like proteins	16.93	0.0000	0.0000
GLEAN_07316	cytochrome P450 6BQ7	15.61	0.0000	0.0000
GLEAN_14090	ABC transporter	13.89	0.0000	0.0006
GLEAN_03745	cytochrome P450 345D2	13.05	0.0000	0.0000
GLEAN_14089	ABC transporter	12.90	0.0001	0.0134
GLEAN_00495	serine protease P8	10.99	0.0000	0.0000
GLEAN_07952	Leucine-rich repeats protein	10.77	0.0001	0.0192
GLEAN_14154	similar to Inter-alpha-trypsin inhibitor heavy chain H4 precursor	10.18	0.0000	0.0000
GLEAN_06593	small heat shock protein 21 isoform 1	10.17	0.0000	0.0116
GLEAN_00542	phosphoserine aminotransferase	9.51	0.0000	0.0001
GLEAN_05026	Short-chain alcohol dehydrogenase	9.26	0.0003	0.0421
GLEAN_00249	serine protease H4	9.15	0.0000	0.0002
GLEAN_15563	apolipoprotein D	8.72	0.0000	0.0008
GLEAN_16089	similar to NRF-6 and NDG-4	8.67	0.0000	0.0007
GLEAN_13326	serine protease P140	8.33	0.0001	0.0147
GLEAN_07322	cytochrome P450 6BQ12	8.19	0.0000	0.0024
GLEAN_05365	similar to cytochrome P450 monooxygenase	8.16	0.0001	0.0172
GLEAN_00497	serine protease P10	8.01	0.0000	0.0088
GLEAN_10252	cytochrome P450 6BK5	7.92	0.0002	0.0280
GLEAN_03029	Protein Kinase	7.91	0.0000	0.0087
GLEAN_12641	Tetraspannin	7.35	0.0000	0.0116
GLEAN_06793	similar to small heat shock protein 21	7.31	0.0000	0.0120
GLEAN_13280	serine protease P139	7.22	0.0000	0.0088
GLEAN_05669	Leucine rich repeat protein	7.10	0.0000	0.0008
GLEAN_15550	similar to cytochrome P450 monooxygenase	7.08	0.0001	0.0149
GLEAN_02431	similar to Ofd1 protein	6.68	0.0000	0.0019
GLEAN_02081	hypothetical protein	6.65	0.0000	0.0015
GLEAN_14157	similar to inter-alpha (globulin) inhibitor H4 (plasma Kallikrein-sensitive glycoprotein)	6.64	0.0000	0.0015
GLEAN_08826	hypothetic al protein	6.58	0.0001	0.0182
GLEAN_11075	Leucine-rich repeat (LRR) protein	6.17	0.0001	0.0182
GLEAN_07911	similar to Glycine N-methyltransferase	6.05	0.0000	0.0087
GLEAN_09457	Leucine-rich repeat (LRR) protein	5.81	0.0000	0.0067
GLEAN_06255	serpin peptidase inhibitor 24	5.79	0.0002	0.0348
GLEAN_15180	Haemolymph juvenile hormone binding protein like	5.75	0.0002	0.0366
GLEAN_14314	leucine-rich repeat receptor-like protein kinase XP_966329.1	5.69	0.0003	0.0474
GLEAN_15436	Major Facilitator Superfamily Transporter	5.67	0.0002	0.0280
GLEAN_09551	arrestin 2	5.64	0.0001	0.0182
GLEAN_15572	similar to Inter-alpha-trypsin inhibitor heavy chain H4 precursor	5.61	0.0001	0.0228
GLEAN_08483	hypothetical protein	5.46	0.0001	0.0142
GLEAN_10766	similar to dual specificity phosphatase 10	5.40	0.0002	0.0407
GLEAN_15379	putative fatty acyl-CoA reductase CG5065-like	5.35	0.0001	0.0255
GLEAN_09696	putative Esterase	5.24	0.0001	0.0182
GLEAN_08299	hypothetical protein	5.08	0.0002	0.0308
GLEAN_04438	toll-like Receptor, Toll3	5.03	0.0001	0.0182
GLEAN_08275	Leucine-rich repeat (LRR) protein	5.01	0.0001	0.0255
GLEAN_16355	similar to glucose dehydrogenase	4.93	0.0001	0.0221
GLEAN_03797	similar to Multi drug resistance 50 CG8523-PA	4.90	0.0001	0.0221
GLEAN_07869	serpin peptidase inhibitor 26	4.78	0.0001	0.0258
GLEAN_06454	similar to lysosomal alpha-mannosidase	4.29	0.0003	0.0494
GLEAN_03391	pleiotrophin-like protein	0.17	0.0001	0.0202
GLEAN_15266	putative esterase	0.14	0.0000	0.0088
GLEAN_13657	haemolymph juvenile hormone binding protein like	0.13	0.0002	0.0366

Of note, we found that several genes encoding proteins with haemolymph juvenile hormone binding domains were significantly induced (e.g. Glean 09776 and 09775) while expression of a paralogous gene was significantly reduced (Glean 13657) upon immune-challenge. These homologues genes may regulate beetle developmental processes by influencing hormone levels. In agreement with this assumption, recent studies described significantly elevated metamorphosis rates [27] or accelerated aging rates [28] in immune-challenged beetles Two further significantly down-regulated genes encode proteins with one an esterase-domain and the other a heparin-binding domain both with unknown function. A deeper understanding of the molecular regulation of beetle development by immune responses would help to unravel potential ecological traits in *Tribolium* that might be traded-off with immune reactions probably similar as shown for other insects [29]–[32].

### Expression rates of immune-related genes upon LPS-challenge in *T. castaneum*

The expression rates of numerous immune-related genes showed high induction levels, such as in the case of attacins and defensins (Table 4). However, due to the limitation of the present in-depth sequencing and calculation procedure, we observed statistical significance in immune-induced expression for only a limited number of immune-related genes (Table 3); short gene sequence and low expression rates of e.g. antimicrobial peptides in naïve animals resulted in a higher variance estimate and a lower confidence in the base mean estimates. Hence, only genes expressed both at medium or high rate and with at least more than 4 fold expression changes were identified by our approach (Figure 3). Particularly genes encoding antimicrobial peptides such as attacins or defensins are expressed at very low level in unchallenged beetles resulting in a high variance estimate in the present analysis resulting in much lower power of statistical analysis. To identify even more genes with significant expression difference a much higher coverage and more replicate determination per treatment with at least 3-fold deeper sequencing [33] would be needed. However, here we will compare tendencies of gene expression changes in immune-challenged *T. castaneum* with reported values of orthologous genes investigated in other insects.

**Table 4:** Expression levels of immune-related genes in adult beetles.

Glean number	Description	log2 (Control)	log2 (LPS-challenge)	Fold change of expression
Microbial recognition				
GLEAN_02546	PGRP-LD	3.5850	3.1699	same*
GLEAN_02789	PGRP-LA	5.8580	6.5699	1.637 up
GLEAN_02790	PGRP-LC	6.9307	8.9915	4.172 up
GLEAN_10508	PGRP-LE	4.5236	4.9542	same
GLEAN_10611	PGRP-SA	3.5850	4.9542	2.583 up
GLEAN_13620	PGRP-SB	no hit	5.169925	n.d.
GLEAN_15689	PGRP-LB	4.3923	5.4919	2.142 up
GLEAN_02295	βGRP1	8.0928	8.6036	same
GLEAN_11529	βGRP2	8.5584	8.4346	same
GLEAN_03991	βGRP3	6.2668	8.0499	3.441 up
GLEAN_14664	TEP-B	7.8074	9.0553	2.375 up
GLEAN_09667	TEP-C	6.9425	7.6935	1.682 up
GLEAN_09375	TEP-A	9.5565	9.3152	same
GLEAN_00808	TEP-D	6.9425	6.5078	same
GLEAN_01981	LpR2	9.8471	10.0954	same
Toll-signaling pathway				
GLEAN_00520	spz1	5.7004	4.9542	1.677 down
GLEAN_01053	spz7	1.0000	3.3219	5.000 up
GLEAN_01054	spz2	4.4594	5.3219	1.818 up
GLEAN_05940	spz3	7.2095	6.9542	same
GLEAN_06726	spz4	no hit	no hit	n.d.
GLEAN_13304	spz5	3.5850	2.5850	2.000 down
GLEAN_16368	spz6	4.6439	3.8074	1.785 down
GLEAN_00625	Toll9	no hit	0	n.d.
GLEAN_00176	Toll1	5.5850	6.3219	1.666 up
GLEAN_04438	Toll3	8.8486	11.2003	5.104 up
GLEAN_04439	Toll4	6.7279	8.7381	4.028 up
GLEAN_04452	Toll2	7.4179	8.3487	1.906 up
GLEAN_04474	Toll7	7.3923	7.5622	same
GLEAN_04895	Toll6	5.3923	4.0875	2.470 down
GLEAN_04898	Toll8	3.3219	4.0875	1.700 up
GLEAN_04901	Toll10	3.5850	3.0000	same
GLEAN_03185	Myd88	7.6865	8.5812	1.859 up
GLEAN_11895	Tube	8.3443	8.4878	same
GLEAN_15365	pelle	6.5078	7.2095	1.626 up
GLEAN_02003	cactus	9.1649	9.7566	1.506 up
GLEAN_09672	pellino	9.9009	9.5981	same
GLEAN_07706	Traf	7.4757	7.8138	same
GLEAN_08782	cactin	8.6439	8.6760	same
GLEAN_07697	Dif1	9.2854	9.2738	same
GLEAN_08096	Dif2	5.8329	6.5850	1.684 up
IMD-signaling pathway				
GLEAN_10851	IMD	4.6439	6.1699	2.880 up
GLEAN_14042	FADD	5.2854	5.3923	same
GLEAN_00068	Dredd	4.4594	5.7004	2.363 up
GLEAN_05572	TAK1	8.8518	8.2668	same
GLEAN_01419	IKKb	7.0000	7.7279	1.656 up
GLEAN_09798	IKKb	11.0934	11.5018	same
GLEAN_00541	IKKg	9.2288	9.8345	1.521 up
GLEAN_11191	REL1	9.7549	10.2228	same
GLEAN_14708	REL2	10.5981	10.2992	same
GLEAN_01189	IAP2	7.8887	7.8202	same
GLEAN_01192	IAP1	9.1085	10.5708	2.755 up
GLEAN_09848	IAP3	9.8281	9.6653	same
GLEAN_02709	IAP4	3.1699	3.5850	same
JAK/STAT-signaling pathway				
GLEAN_01874	domeless	9.59246	10.1762	same
GLEAN_13218	STAT92E	10.6909	10.3487	same
GLEAN_08648	hopscotch	7.2668	7.0980	same
JNK-signaling pathway				
GLEAN_00385	hemipterous	6.2479	6.9887	1.671 up
GLEAN_06810	basket	8.4959	8.8642	same
GLEAN_06814	D-Jun	7.7279	8.7549	2.037 up
GLEAN_10766	Puckered	6.3576	8.8106	5.475 up
Effector molecules				
GLEAN_07737	attacin1	0.0000	4.3219	20.000 up
GLEAN_07738	attacin2	0.0000	4.7549	27.000 up
GLEAN_07739	attacin3	no hit	0	n.d.
GLEAN_06250	defensin1	no hit	2.3219	n.d.
GLEAN_10517	defensin2	0.0000	3.8074	14.000 up
GLEAN_12469	defensin3	no hit	no hit	n.d.
GLEAN_00517	thaumatin1	5.0875	9.1898	17.176 up
GLEAN_11564	thaumatin2	5.7004	5.2479	same
GLEAN_00515	thaumatin3	8.1241	7.7279	same
GLEAN_00516	thaumatin4	4.8580	3.5850	2.416 down
GLEAN_00518	thaumatin5	no hit	0	n.d.
GLEAN_10349	lysozyme1	3.3219	no hit	n.d.
GLEAN_10350	lysozyme2	0.0000	2.3219	5.000 up
GLEAN_10351	lysozyme3	2.8074	4.8580	4.142 up
GLEAN_10352	lysozyme4	5.0444	6.1293	2.121 up
Stress-related immune-responsive genes				
GLEAN_15563	apoD	6.4919	9.6366	8.844 up
GLEAN_10172	Hsp68	4.9069	10.5934	51.500 up
GLEAN_05338	Hsp27	1.5850	7.0000	42.666 up

n.d., not determinable;

* same, genes with expression difference less than 1.5 fold.

### Molecular pattern recognition proteins

Peptidoglycan recognition proteins (PGRPs) are evolutionarily conserved in animals and have been found to bind specifically to and to hydrolyze bacterial peptidoglycan. In addition, peptidoglycan-bound insect PGRPs activate the Toll and IMD signal transduction pathways as well as immune-related proteolytic cascades [4], [5]. Genome-wide gene expression profiling of the *Drosophila* immune-response implied that five PGRP genes including PGRP-SA, SC2, SB1, LB and SD are up regulated upon septic injury [6]. Here, we found that 5 of 7 *Tribolium* genes encoding PGRP- SA, LA, LC, SB, and LB were up regulated in response to LPS injection whereas the expression rates of PGRP-LE and LD were not significantly influenced. In *Drosophila*, PGRP-LD is only expressed in hemocytes and its function is yet unknown whereas PGRP-LE is an intracellular receptor capable of binding bacteria in the cytoplasm [4].

Gram negative bacteria binding proteins (GNBP) comprises a family of proteins, also known as β-1,3-glucan binding proteins (βGBP) and β-1,3-glucan recognition proteins (βGRP). The first β-1,3-glucan recognition protein was purified from the hemolymph of the silkworm *Bombyx mori* with a strong affinity for gram negative bacteria [34]. This GNBP contained a region with significant sequence homology to the catalytic region of a group of bacterial β-1,3-glucanases. In *Drosophila*, three GNBP paralogs (GNBP1, GNBP2 and GNBP3) are known, which only GNBP3 (CG13422) is immune-responsive upon septic injury [4]. GNBP1 is required for Toll activation in response to gram positive bacterial infection whereas GNBP3 has been reported to sense fungal infections [4]. The biological function of *Drosophila* GNBP2 has yet not been determined. In *Tribolium*, we found up regulation of βGRP3 but not of βGRP1 and βGRP2 upon LPS-challenge, which resembles observations from *Drosophila*.

Thioester-containing proteins (TEPs) are a further group of bacteria-binding proteins, which function as both opsonins and protease inhibitors [4], [5]. In *Drosophila*, TEP II and TEP IV of in total 6 paralogous TEPs were found to be induced upon septic injury [6]. In *T. castaneum* only 4 TEPs are traceable in the whole genome sequence and in this study, we found that mRNA levels of *Tribolium* TEP-B and C were increased but not of TEP-A and D upon immune-challenge. Interestingly, no clear orthologs can be assigned between dipteran and coleopteran TEPs, except for *Tribolium* TEP-A, which is orthologous to *Drosophila* TEP-VI [11]. Finally, a putative TEP/complement-binding receptor-like protein (LpR2) was shown to be immune-inducible in *Drosophila* but failed to exhibit significant difference in gene expression rates in *Tribolium* in our approach.

### Immunity related signaling

In insects, major immune-related signaling pathways include Toll, IMD, JAK-STAT, and JNK pathways [4]. Mammalian Toll-like receptors are capable of directly binding danger (e.g. extracellular nucleic acids or uric acid) or pathogen-derived molecules (e.g. LPS) while *Drosophila* Toll is instead activated by a proteolytically activated cytokine-like molecule spaetzle 1. In *D. melanogaster* spaetzle 1 is immune-responsive [6] and five further paralogs have been described (spaetzle 2–6) with yet unknown functions. In *Tribolium*, 7 spaetzle paralogs exist with spaetzle 3, 4, 5, and 6 representing orthologs to respective *Drosophila* spaetzle isoforms. However, no *Tribolium* ortholog of *Drosophila* spaetzle 2 can be found and spaetzle 1, 2 and 7 in *Tribolium* form a clade together with the single, immune-responsive *Drosophila* spaetzle 1 [11]. Here, we found that *Tribolium* spaetzle 2 and 7 are immune-inducible, whereas spaetzle 1 was immune-repressed.

Similarly to their potential ligands, also Toll-like receptors have experienced lineage-specific gene duplications in beetles as well as in flies or mosquitoes [11]. 4 *Tribolium* Toll-like receptors (1 to 4) of in total 10 paralogs were described to form a clade with the single, immune-responsive *Drosophila* Toll receptor of the in total 9 *Drosophila* Toll-like receptors [11]. Here, we observed that these *Tribolium* Toll-like receptors 1 to 4 were induced in their gene expression upon LPS-challenge similar to the *Drosophila* Toll receptor upon septic injury [6]. In addition, *Tribolium* Toll6 was over 2-fold down-regulated whereas Toll8 was slightly upregulated. Taken together, these results support the hypothesis that *Tribolium* has a more complex immune-related Toll signaling than *Drosophila*, since both a higher number of immune-responsive Toll-like receptors and of spaetzle ligands exist in *Tribolium* than in *Drosophila*.

Regarding further immune-related signaling pathways, we found 2 to 5 fold induced expression of several signaling proteins involved in IMD and JNK pathways such as IMD and D-Jun, respectively, which is in agreement to observations from *Drosophila* [6]. Also in agreement with observations in *Drosophila* [6], we found that expression rates of JAK-STAT pathway genes were not significantly influenced by LPS-challenge in *Tribolium*.

### Antimicrobial peptides

As expected, we identified genes encoding antimicrobial peptides such as defensins, attacins and thaumatin among the systemically most septic injury inducible genes with up to 10 to 30 fold higher expression rates in LPS-challenged animals than in naive ones. This is in agreement with observations from diverse immune-challenged invertebrates [6], [7], [9], [35]–[37].

### Stress response genes

Recently, we determined induced expression of genes in *T. castaneum* involved in detoxification and stress adaptation such as apolipoprotein D, cytochrome P450, gluthathione S-transferase, and a number of heat shock proteins [12]. In line with these observations, here we found elevated gene expression rates of a number of stress and detoxification genes upon LPS-challenge including most notable HSPs, CytP450s (e.g. 6BQ7, 345D2, 6BQ12, 6BK5), GST, ApoD, and ABC transporters (Table 3). This supports our recent hypothesis that interdependencies between immune and stress responses exist in *T. castaneum* [12], [38].

It should be noted here that wounding itself can lead to gene expression alterations in insects triggered by e.g. cryptic, endogenous danger signals such as nucleic acids or collagen fragments [39], [40]. Moreover, the presently used LPS preparation is known to include bacterial nucleic acids and peptidoglycans, which may be responsible for the induction of e.g. PGRPs and PGRP-controlled genes. Hence, in follow-up studies we propose to investigate transcriptomic immune responses from beetles with varying treatments such as feeding and stabbing with different elicitors and pathogens of diverse phylogenic origin and much more time points of samples derived from whole animals, specific organs, tissues or cell types. In addition, different *T. castaneum* genotypes, sexes or developmental stages are likely to vary in their immune investment and hence may show altered gene expression upon immune-challenge, particularly in the context of diverse environmental cues and stresses.

## Conclusions

The beetle immune response underlies the differential expression of a wide array of different genes. Here we describe differential expression of numerous immune-related genes as well as several genes encoding proteins with leucine-rich-repeat domains, which might function as receptors in specific immune recognition and signaling reactions in beetles maybe in a similar way as leucine-rich-repeat domain containing receptors in ancient jawless vertebrates [41]. While insect immune defense mechanisms had generally been assumed to be non-specific, diverse insects including the red flour beetle *T. castaneum* have recently been shown to respond quite specifically to some pathogens [42]–[45]. Presently identified genes may help to elucidate the molecular basis of such specific reactions. This study is the first whole transcriptome analysis of the complex gene expression response in *T. castaneum* upon septic injury and provides numerous candidate genes that we can use as a starting point for further studies on beetle immunity.

## Supporting Information

Data S1 *De novo* assembly of Illumina reads.
(ZIP)

Figure S1 Quality score boxplot drawing of the Illumina sequencing reads.
(PDF)

Table S1 List of high-quality SNPs and DIPs within the coding gene sequences between the *T. castaneum* strain San Bernardino used in the present analysis and the genome-sequenced strain Georgia GA-2.
(CSV)

Table S2 DESeq analysis of transcriptome sequencing analysis derived by fitting normalized count data with a generalized linear model (GLM) estimating a negative binomial distribution to the calculated mean values of the two biologically independent samples, fold changes and respective P values (pval) as well as P values adjusted (padj) for multiple testing with the Benjamini-Hochberg procedure, which controls false discovery rate (FDR).
(TXT)

Table S3 Blast2go annotation of predicted genes (gleans) to assign the potential functions of identified genes.
(XLSX)
