# Supplementary figures and images for: Next Generation Sequencing Based Transcriptome Analysis of Septic-Injury Responsive Genes in the Beetle Tribolium castaneum
Source: PLoS One. 2013 Jan 9;8(1):e52004. doi: 10.1371/journal.pone.0052004 (PMC3541394; doi:10.1371/journal.pone.0052004)

Quartiles 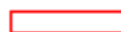  
Medians 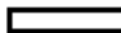  
Outliers 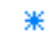

Box plot in Galaxy

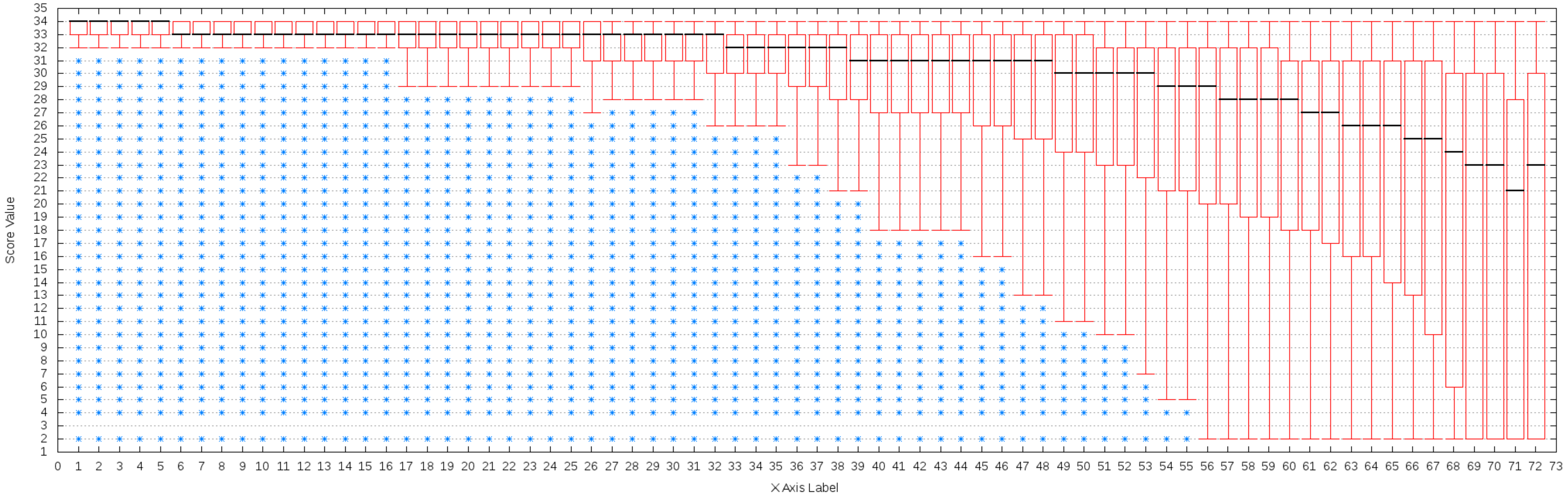

Supplement: Figure S1 — Quality score boxplot drawing of the Illumina sequencing reads. (PDF) [file pone.0052004.s002.pdf]
